# Lifestyle-Driven Variations in Nutrimiromic MicroRNA Expression Patterns across and beyond Genders

**DOI:** 10.3390/life14030390

**Published:** 2024-03-15

**Authors:** Angelika Pointner, Ulrike D. B. Krammer, Elena Tomeva, Ulrich Magnet, Berit Hippe, Ursula Jacob, Alexander G. Haslberger

**Affiliations:** 1 Department of Nutritional Sciences, University of Vienna, 1090 Vienna, Austria; angelika.pointner@univie.ac.at (A.P.); uk@healthbiocare.at (U.D.B.K.); berit.hippe@univie.ac.at (B.H.); 2 HealthBioCare GmbH, 1090 Vienna, Austria; et@healthbiocare.at (E.T.); ulrich.magnet@healthbiocare.at (U.M.); 3 Dayclinic Dr. Jacob, 81675 Munich, Germany; info@doc-jacob.com

**Keywords:** epigenetic, miRNAs, nutrition, lifestyle, biomarker, nutrimiromics, precision nutrition, smoking, inflammation

## Abstract

The importance of diet and lifestyle in maintaining overall health has long been recognised. MicroRNAs (miRNAs) have emerged as key players in the intricate interplay between health and disease. This study, including 305 participants, examined the role of miRNAs from capillary blood as indicators of individual physiological characteristics, diet, and lifestyle influences. Key findings include specific miRNAs associated with inflammatory processes and dietary patterns. Notably, miR-155 was associated with subjects with metabolic diseases and upregulated in age. Additionally, the study revealed diet-related miRNA expressions: high consumption of vegetables, fruits, and whole grains correlated with increased levels of miR-let-7a and miR-328, both implicated in anti-inflammatory pathways, and decreased expression of pro-inflammatory miR-21. In the context of smoking, we found a significant decrease in miRNA-142, known for its downregulation in lung cancer. We observed a sex-biased expression of various miRNAs with significant upregulation of miR-151a in females and a higher expression of miR-155 in ageing females, representing a possible mechanism for the increased susceptibility to autoimmune diseases. In conclusion, the study underscores the significant influence of lifestyle, nutrition, and sex on miRNA profiles. Circulating miRNAs demonstrate significant potential as biomarkers in personalized medicine, highlighting their utility in tailoring healthcare to individual needs.

## 1. Introduction

Nutrition and lifestyle modifications have long been recognized as pivotal factors in the realm of disease prevention and the promotion of healthy ageing. However, in the dynamic field of personalised medicine, precision nutrition is emerging as a key component that is revolutionising our approach to health promotion and disease prevention [1,2,3]. By tailoring nutritional recommendations to an individual’s genetic predisposition, metabolic profile, microbiome, and health status as well as environmental influences and lifestyle habits, precision nutrition is at the forefront of fostering optimal health outcomes and enabling healthy ageing [3,4]. Given the comprehensive approach of precision nutrition, the importance of sex and gender within this context is increasingly acknowledged. It becomes more and more evident that sexual dimorphism is apparent in a range of physiological functions as well as pathological conditions and, in particular, affects both innate and adaptive immune responses [5,6,7]. Scientific progress in understanding the mechanisms behind dietary interventions, as well as the effects of lifestyle choices, has been greatly enhanced by the integration of metabolomics, proteomics, and, more recently, metagenomics [8]. While genome-wide association studies have unveiled the genetic basis of not only numerous disorders but also sex-specific genetic variance [9,10], epigenetic modifications such DNA methylations, histone modifications, or non-coding RNAs have provided a deeper understanding of physiological processes and disease prevention.

With advances in sequencing technologies, microRNAs (miRNAs) have attracted particular interest among epigenetic modifications. These small, non-coding RNAs, typically 20–22 nucleotides in length, play a significant role in regulating gene expression at both the transcriptional and post-transcriptional levels [11,12]. After biogenesis in the nucleus and further processing in the cytoplasm, either the 3p or the 5p end of the mature miRNA binds to Argonaute protein and forms a ribonucleoprotein effector complex, known as the RNA-induced silencing complex (RISC). Accordingly, the endings 3p and 5p in the miRNA nomenclature refer to strands derived from the 3′ and 5′ ends of the precursor molecule, indicating their origin and their potentially different roles in gene regulation. RISC targets complementary messenger RNAs (mRNAs), usually resulting in either translational repression or endonucleolytic degradation [13,14]. By targeting numerous mRNAs, each miRNA can influence a broad spectrum of physiological and pathological processes, emphasising their importance as key regulators of cell function [15]. Moreover, miRNAs are known to contribute to both innate and adaptive immune responses and are involved in posttranscriptional feedback control mechanisms participating in the regulation of metabolism and inflammation [16,17]. Considering their essential biological role, it comes as no surprise that the expression of miRNAs is rigorously regulated and an aberrant expression of miRNAs is associated with a wide range of diseases including cancer, neurodegenerative disorders, diabetes, obesity, and related conditions, as well as autoimmune processes [18,19,20,21,22]. However, a number of investigations have substantiated the capacity of miRNAs to undergo modulation in response to dietary, lifestyle, and environmental factors [23,24,25,26,27]. Given these reasons, miRNAs are emerging as promising sensitive prognostic, diagnostic, and theragnostic biomarkers reflecting the health status of the body, especially those found circulating in various bodily fluids such as human plasma [18,28]. They not only offer ease of acquisition but also occur in a particularly stable form here [29].

The main objective of the present study was to investigate how lifestyle factors such as diet, exercise, and tobacco and alcohol consumption affect miRNA expression patterns differently in men and women. In addition, we wanted to assess the impact of these lifestyle choices as a function of individual health status, focussing in particular on metabolic disorders such as diabetes and obesity. Therefore, the aim of our study was to determine whether the effects of lifestyle on miRNA profiles vary not only by gender but also by participants’ metabolic status to provide a comprehensive understanding of the complex interactions between diet, lifestyle, and specific miRNAs in health and disease, while also highlighting potential avenues for future research. As a non-invasive method, we used capillary blood samples, which provide a convenient and accessible way to obtain valuable diagnostic information.

In our study, we carefully selected 27 miRNAs to investigate a spectrum of established and emerging miRNAs that are primarily associated with inflammation, cancer, metabolic disorders, and adipogenesis. The selection includes well-known families such as the let-7 family and the miR-15a/16 cluster, known for their role in inflammation and tumour control, as well as miR-21, miR-146b, and miR-155, which have been highlighted in relation to inflammageing [30,31,32,33,34]. Furthermore, we also considered less-investigated miRNAs with specific tissue expressions and a suggested potential as disease biomarkers, such as miR-122 for metabolic syndrome and type 2 diabetes and miR-30 for heart disease [35,36]. With this approach, we aim to deepen the understanding of miRNA functions in health and disease while exploring the interaction between dietary patterns and miRNA expression.

## 2. Materials and Methods

### 2.1. Study Group

The study included a total of 305 participants, comprising 197 healthy controls with no prior medical conditions and a BMI below 30 and 108 individuals with mild metabolic disorders. Table 1 presents demographic and health characteristics of the study participants categorized into “healthy” and “metabolic disease” groups. The latter consisted of individuals with well-controlled metabolic conditions, such as thyroid dysfunction (44.4%), obesity (34.3%), and type 1 or 2 diabetes (10.2%), among others (11.1%). Exclusion criteria encompassed pre-existing medical conditions such as acute infections including common colds, flu-like symptoms, all types of cancerous diseases, and severe cardiovascular disorders, specifically coronary heart disease, cerebrovascular disease, peripheral arterial disease, rheumatic heart disease, congenital heart disease, deep vein thrombosis, and pulmonary embolism. The differences in the number of test subjects in some results are due to the fact that not all participants in the study answered all the questions in the questionnaire.

### 2.2. Questionnaire

Participants were required to complete a comprehensive questionnaire that included not only dietary habits as part of a well-established food frequency questionnaire (FFQ) but also lifestyle factors such as physical activity, exercise, alcohol consumption, and smoking.

### 2.3. Sample Collection

Capillary blood was obtained from participants’ fingertips with safety Lancets Extra 18 G (Sarstedt, Nümbrecht, Germany) and collected utilizing Whatman^®^ protein saver cards (Sigma-Aldrich, Vienna, Austria), followed by overnight drying. From each dried blood spot (DBS), two circular sections measuring 10 mm in diameter were extracted. Subsequently, RNA isolation was carried out using the MagMAX™ FFPE DNA/RNA Ultra Kit (Thermo Fisher, Vienna, Austria) following the manufacturers’ protocol. The collected samples were then stored at a temperature of −20 °C.

### 2.4. cDNA-Synthesis and Real-Time PCR (qPCR)

For miRNA analysis, cDNA synthesis was conducted using the TaqManTM Advanced miRNA cDNA Synthesis Kit (A28007; provided by Applied BiosystemsTM through Thermo Fisher Scientific) and a SimpliAmpTM Thermal Cycler (provided by Applied BiosystemsTM through Thermo Fisher Scientific). After completion, the cDNA samples were preserved at a temperature of −20 °C until further analysis. TaqManTM Advanced miRNA assays was used under the default settings on QuantStudioTM 3 from Thermo Fisher, Landsmeer, Netherlands. To mitigate technical variability and guarantee data reliability, we incorporated technical replicates. Expression was examined using miR-24-3p and miR-93-5p as reference genes using the Delta CT (ΔCT) method. These particular miRNAs are recommended as normalizers especially for serum and plasma, as they show stable expression there [37]. ΔCT was calculated as the difference between the cycle thresholds (CT) of the target gene and the reference gene and serves as a relative measure of gene expression.

In this study, we profiled a set of 27 miRNAs, which included miR-let-7a-5p, miR-let-7g-5p, miR-10a-5p, miR-15a-5p, miR-16-5p, miR-19b-3p, miR-21-5p, miR-26b-5p, miR-29c-3p, miR-30e-3p, miR-30e-5p, miR-101-3p, miR-106b-5p, miR-122-5p, miR-126-3p, miR-127-3p, miR-132-3p, miR-139-5p, miR-142-3p, miR-146a-5p, miR-150-5p, miR-151a-5p, miR-155-3p, miR-181a-5p, miR-328-3p, miR-378a-3p, and miR-877-5p. For readability, the specific 3p and 5p suffixes are omitted from the main text.

### 2.5. Statistical Analysis

The statistical analysis was conducted utilizing IBM SPSS Statistics 29 and GraphPad Prism 6 software. Paired *t*-tests and ANCOVA (adjusted for baseline) were employed to compare sex-specific expression of miRNAs. Linear regression models, with adjustments for covariates, were used to analyse effects of age and BMI on miRNA expression. Nonparametric Kruskal–Wallis test with a post hoc Dunn–Bonferroni test was used for observing differences across BMI groups. Associations between miRNA expressions and lifestyle factors were examined through ANCOVA. Distribution of potential confounders age, BMI, smoking behaviour, and frequency of sports was similar across categories of alcohol consumption as determined by one-way ANOVA and Pearson’s Chi-square test. A significance threshold of *p* ≤ 0.05 was applied for all analyses. In case of missing data points, missingness analysis was performed using Little’s missing completely at random (MCAR) test. For MCAR conditions, we predominantly opted for complete case analysis to minimize bias and maintain data integrity in our miRNA lifestyle factor analysis.

## 3. Results

### 3.1. Age-Related Variations in MiRNA Expression

**Figure 1:**
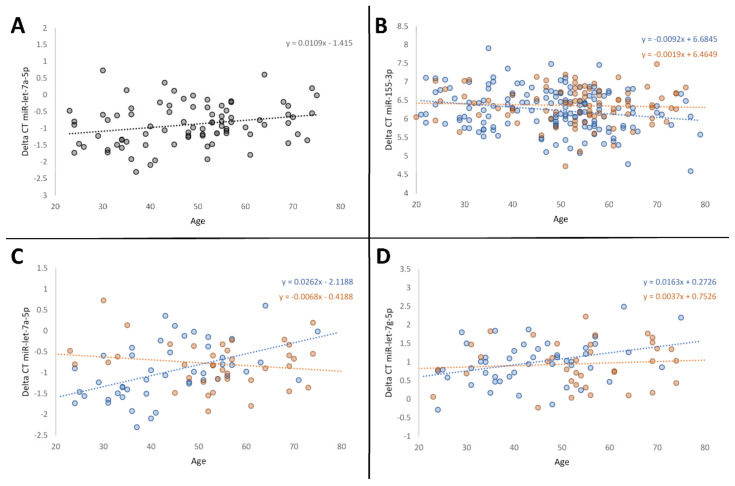
Linear regression analysis between microRNA expression quantified by delta CT and age. Panel (**A**) describes age specific alterations in miR-let-7a including the whole sample group; panels (**B**–**D**) show observations for miR-155, miR-let-7a, and miR-let-7g separated for the subgroups, symbolized in blue (healthy controls) and orange (metabolic disease group). Delta CT values are inversely correlated to miRNA expression.

After the adjustment for potential confounders using linear regression models, decreasing expression of miR-let-7a was significantly associated with age (95% confidence interval = 0.001–0.021, *p* = 0.027). In healthy controls, an age dependent decrease in miRNA expression was found in miR-151a, miR-let-7a, and miR-let-7g (95% confidence interval = 0.005–0.032, *p* = 0.008; 0.016–0.043, *p* < 0.001; 0.006–0.031–0.02, *p* = 0.006) (Figure 1). A significant increase with age was found for miR-155 (95% confidence interval = (−0.016)–(−0.003, *p* = 0.005)), and a trend in the same direction could be observed for miR-19b. Age-dependent expression of miRNAs in the metabolic disease group appeared completely detached from healthy controls. No significant age dependent changes in expression could be seen here. The examination of sex-based differences in expression with ageing revealed a significant higher expression of miR-155 in ageing females (Coefficient = −0.075, *p* = 0.010).

### 3.2. BMI Effect on MiRNA Levels

**Figure 2:**
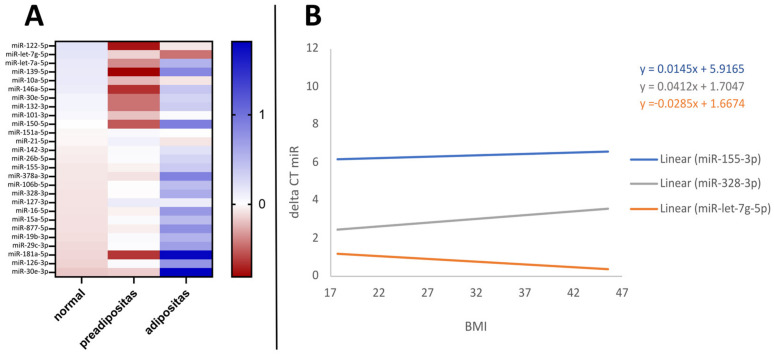
(**A**) Heatmap showing differential microRNA expression z-scores across BMI groups as generally classified by the World Health Organization (WHO) for normal 18.5–24.9 kg/m^2^, preadipositas 25–29.9 kg/m^2^, and adipositas >30 kg/m^2^). Z-scores are computed by subtracting the mean ΔCT value of all samples from each individual ΔCT value and dividing by the standard deviation, normalizing expression data for comparison. Red and blue shadings represent higher and lower expression levels, respectively. (**B**) Linear regression analysis between microRNA expression (delta CT) and BMI shows significant changes for miR-155, miR-let-7g, and miR-328. Delta CT values are inversely correlated to microRNA expression.

For analysis of BMI effects on miRNA expression, adipose probands from the metabolic group with the absence of any further metabolic diseases were included. BMI analysis was conducted both linearly, evaluating BMI as a continuous variable, and categorically, according to the established BMI groups defined by the World Health Organization (WHO). After adjustment for age and sport frequency using linear regression models, increasing expression of miR-let-7g was significantly associated with BMI (95% confidence interval = −0.093–0.012, *p* = 0.013). A significant decrease with BMI was found for miR-155 and miR-328 (95% confidence interval = 0.007–0.040, *p* = 0.006, 0.013-0.111, *p* = 0.015) (Figure 2B). A nonparametric Kruskal–Wallis test with a post hoc Dunn–Bonferroni test was used for observing differences across BMI groups as generally classified by the WHO for underweight <18.5 kg/m^2^, normal 18.5–24.9 kg/m^2^, preadipositas 25–29.9 kg/m^2^, and adipositas >30 kg/m^2^. Distribution of age in years was the same across BMI groups from normal to adipose, and sport frequency was considered as confounder. Several miRNA, including miR-155, miR-30e, miR-378a, miR-15a, miR-16, miR-26b, miR-106, miR-126, miR-328, and miR-877, showed a trend towards lower expression associated with overweight and/or adipositas groups. MiR-let-7g and miR-21 were positively associated with higher BMI groups but were also not significant (Figure 2A). This specific study population showed no statistically significant difference in BMI between male and female participants (*p* = 0.931). However, significant findings in female miRNA expression showed a decrease for miR-155 and miR-378a, and an increase for miR-let-7g, with corresponding *p*-values of 0.002, 0.047, and 0.012 after adjusting for age and sport, while there were no significant changes noted in males.

### 3.3. MiRNA Expression across Different Health Statuses

The 108 people in the study subgroup with mild metabolic disorders were older on average and had a higher BMI than the 197 healthy control subjects. An analysis of covariance (ANCOVA) was performed with age and BMI as covariates to evaluate differences between the two subgroups. The analysis revealed a significant lower expression of miR-155 in persons with mild metabolic disorders (*p* = 0.047), while other trends were observed but did not reach statistical significance. No significant gender-specific differences were found in miRNA behaviour between the healthy and metabolic disease groups. Our analysis revealed notable differences in miRNA expression between individuals with specific metabolic diseases and those without any metabolic disease. It is important to note that due to the relatively small sizes of some groups, adjustments for age and BMI were not feasible, leading to potential heterogeneity within the groups. In individuals with type 1 diabetes mellitus, significant differences were observed in the expression of miR-378a (*p* = 0.000) and miR-122 (*p* = 0.019), and a strong trend in miR-142 (*p* = 0.075), which all showed lower expression compared to the healthy control group. Individuals with type 2 diabetes mellitus showed a strong negative trend in miR-15a (*p* = 0.067), miR-16 (*p* = 0.075), and miR-155 (*p* = 0.082).

### 3.4. Impact of Smoking Behaviour on MiRNA Expression

FFQ data for smoking behaviour showed that 14.8% were current cigarette smokers, 29.8% were former smokers, and 51.1% were non-smokers. Current smokers were younger than former smokers (*p* = 0.034). Two-factorial ANCOVA with age as covariate was conducted to both isolate the effects of smoking on miRNA expression and assess a potential interaction of smoking behaviour and alcohol consumption. Covering the whole sample group, we observed a significant main effect of smoking on expression of miR-142 (F(2, 76) = 3.81; *p* = 0.027; partial η2 = 0.091) with a medium effect size of f = 0.32, but no significant interaction effect between smoking and alcohol consumption (F(4, 76) = 0.56; *p* = 0.694; partial η2 = 0.028) (Table 2). Bonferroni-corrected post hoc tests showed a decrease in miR-142 expression in the smoking group (Table 2) with a significant difference between former smokers and current smokers (*p* = 0.023). This could be also seen in the healthy subgroup (F(2, 44) = 5.202; *p* = 0.009; partial η2 = 0.191) and the metabolic disease group, but concerning the latter, the effect was not significant. A strong trend could be seen for miR-19b, but it was not significant. Although some further trends were apparent, no other significant differences were found between the sample groups or gender-specific changes in smoking behaviour.

### 3.5. Alcohol Consumption and Effects on MiRNA Expression

**Figure 3:**
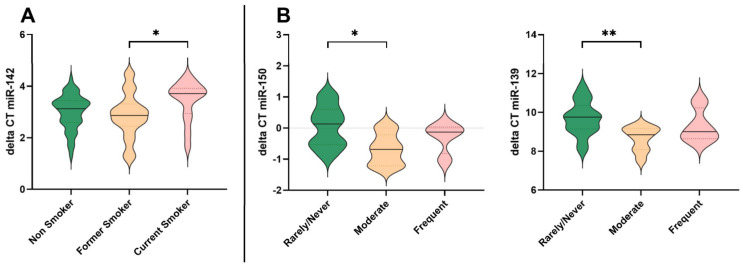
Violin plots showing significant changes in gene expression of miRNA-142, miR-150, and miR-139 in dependence on smoking (**A**) or alcohol drinking behaviour (**B**). MiRNA expression was quantified as delta CT values, inversely correlated to microRNA expression. *p*-values less than 0.05 are summarized with one asterisk and *p*-values less than 0.01 are summarized with two asterisks.

FFQ data for alcohol consumption showed that 46.3%of all participants stated to drink alcohol rarely or never, 38% were moderate drinkers (3–5 times a month), and 15.7% consumed alcohol frequently (5–7 times a week). There was no participant reporting a drinking pattern of multiple times a day. Distribution of potential confounders age, BMI, smoking behaviour, and frequency of sports was similar across categories of alcohol consumption as determined by one-way ANOVA and Pearson’s Chi-square test for the categorical variables (*p* = 0.094, *p* = 0.304, *p* = 0.538, *p* = 0.650). Effect sizes were determined based on ANCOVA adjusted means with age as covariate. Covering all participants, we observed a significant effect of alcohol drinking in miR-150 and miR-139 (F(2, 28) = 5.206; *p* = 0.012; partial η2 = 0.271) and (F(2, 26) = 5.679; *p* = 0.009; partial η2 = 0.304) (Figure 3). Bonferroni-corrected post hoc tests showed a significant increase in expression in miR-150 as well as miR-139 in moderate drinkers compared to non-drinkers (*p* = 0.010, *p* = 0.007). Adjusted means also showed an increase in frequent drinkers, but not significant. No significant sex-biased differences in the effects of alcohol consumption on expression of miRNAs were found.

### 3.6. MiRNA Expression across Different Diet Clusters

A two-step cluster analysis was conducted to identify two subgroups of individuals representing different levels of nutrition behaviour with consideration of health aspects. Therefore, intake of vegetables and fruits, whole grains, and ready meals were set as classification variables. Group 1 had a higher intake of vegetables and fruit as well as whole grains than group 2 and ready meals were not part of their daily diet, whereas in group 2, 41.2% of participants stated to have ready meals multiple times a week. Distribution of sex was similar between the two diet groups (Pearson’s Chi-square test: *p* = 0.649). To assess potential effects of a health-related diet on the expression of miRNAs, ANCOVA between the two diet cluster groups was used with age and BMI as covariates. Interaction effects appropriate to the respective miRNA were investigated. Across the whole sample group, there was a significant higher expression of miR-let-7a (*p* = 0.003) and miR-328 (*p* = 0.045) in diet cluster 1, and a strong trend for miR-877 in the same direction. For miR-let-7a, this could be also observed in both healthy and metabolic disease subgroups (*p* = 0.010; *p* = 0.030). For healthy controls, expression of miR-151a was positively affected by a healthy diet (*p* = 0.040), whereas in the metabolic disease group, miR-877 and miR-21 showed a lower and a higher expression, respectively (*p* = 0.019, *p* = 0.004). No significant interaction effects were found. In women, a significantly higher expression of miRNA-328 was associated with diet cluster 1 (*p* = 0.000), as well as an interaction effect with exercise (*p* = 0.012).

### 3.7. Impact of Milk Consumption on MiRNA Profiles

Distribution of sex, age, and BMI was different among the categories of milk intake (Pearson’s Chi-square test *p* = 0.005, ANOVA *p* = 0.001). To analyse a potential influence of milk consumption on expression of miRNAs, ANCOVA was conducted with BMI as covariate. Interaction terms of milk consumption with sex, meat, vegetables, and fruit intake, and sports activity were assessed. Covering the whole sample group, there was a significant main effect of milk consumption on expression of miR-15a, miR-16, miR-26b, miR-106b, and miR-126. Bonferroni-corrected post hoc tests showed a significant decrease in expression in the group with an everyday consumption of milk compared to rarely/never milk product consumers (*p* = 0.020, *p* = 0.014, *p* < 0.001, *p* = 0.024, *p* = 0.003). Regarding miR-26b, there was a significant interaction effect of milk consumption and sports activity (*p* = 0.021), indicating that a moderate sports activity level of 1–2/week has a decreasing influence on expression of miR-26b in people with low milk consumption. Further analysis of the subgroups showed the same trends for the miRNAs mentioned, although not significant as far as the healthy control group was concerned. Only in the metabolic disease group could a significant main effect on expression be shown for miR-26b and miR-126 (*p* each 0.020).

### 3.8. Effects of Meat Consumption on MiRNA Expression

In the whole sample group, distribution of age was similar among different frequencies of meat consumption (*p* = 0.416). BMI, as well as smoking frequency, showed a significant positive bias with increasing meat consumption (ANOVA *p* = 0.038, Pearson’s Chi-square test *p* = 0.021), and sports activity was negatively associated (Pearson’s Chi-square test *p* = 0.017). Whilst controlling for BMI, ANCOVA showed a significant effect on expression of miR-21, miR-132, miR-16, miR-26b, miR-29c, miR-106b, and miR-126 (*p* = 0.033, *p* = 0.014, *p* = 0.029, *p* = 0.023, *p* = 0.002, *p* = 0.039, *p* = 0.001). Bonferroni-corrected post hoc tests revealed a significant higher expression of miR-132 in daily meat consumers compared to participants who stated to eat meat never or rarely. In contrast, expression of miR-21, miR-16, miR-26b, miR-29c, miR-106b, and miR-126 was lower in daily meat consumers. No interaction terms between meat consumption and sports activity or smoking frequency could be seen. For the healthy and metabolic subgroup, the directional effect on expression was similar, but significance was only seen in the healthy control group.

### 3.9. Impact of Vegetable and Fruit Intake on MiRNA Expression

A lower BMI as well as younger age was significantly associated with a higher intake of vegetable and fruit (ANOVA *p* = 0.001, *p* = 0.035). ANCOVA was conducted with age and BMI as covariate to assess a potential effect of vegetable and fruit intake on miRNA expression. MiR-21 and miR-29c were significantly lower expressed with a higher consumption of vegetable and fruit (*p* = 0.037, *p* = 0.005). An interaction effect of vegetable and fruit intake with sports activity, smoking frequency, or other food groups could not be seen. The analysis of gender subgroups revealed a significant association in the female group between higher consumption of vegetables and fruit and the increasing expression of miRNA-328 (*p* = 0.002).

### 3.10. Sex-Biased MiRNA Expression

**Figure 4:**
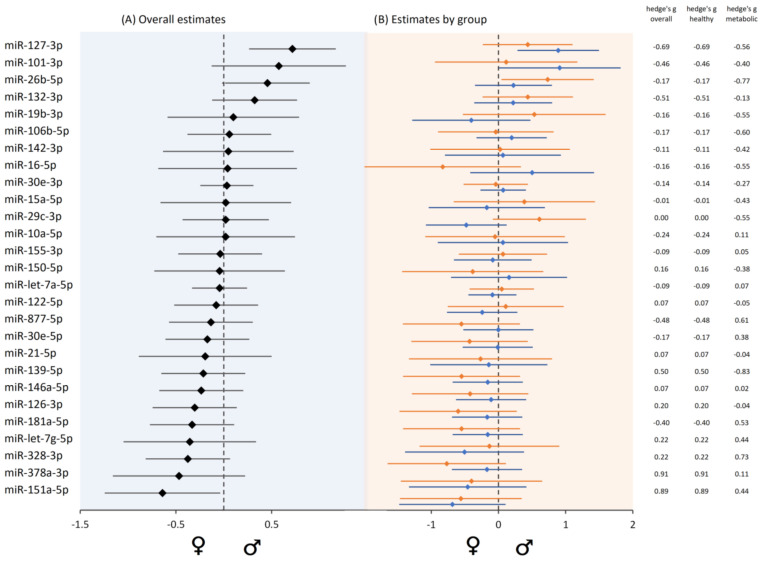
Forest plot showing sex bias in microRNA expression quantified by delta CT values (effect sizes as hedges’ g and the corresponding 95% confidence intervals). (**A**) Overall effect sizes in whole sample group. (**B**) Effect sizes for the two subgroups are displayed separately in blue for healthy controls and orange for the metabolic disease group. Positive estimates mean male bias and negative female bias, the dashed line (zero) means no sex bias.

We used the independent samples *t*-test as well as the Wilcoxon’s test to identify differentially expressed miRNAs between genders. In the whole sample group, several miRNAs showed a directional sex specific trend (Figure 4). However, only miR-151a showed a significant positive bias towards females (*p* = 0.002). Regarding healthy controls, miR-151a was also found to significantly associate with sex (*p* = 0.004). In probands with metabolic disorders, miR-328 was significantly lower expressed in men (*p* = 0.038).

## 4. Discussion

The complex interplay between nutrition, lifestyle choices, and miRNAs is a promising area of research that lies at the nexus of cutting-edge health strategies. In this context, precision nutrition, with its customised interventions based on individual profiles combines synergistically with miRNA research, focusing on the molecular mechanisms underlying health changes. Not all functions of miRNAs are understood, yet their specific roles contribute to biomarkers and innovative therapies [38,39,40]. However, the extent to which circulating miRNAs mirror activities in specific tissues is still under investigation. Nonetheless, several studies have shown that the patterns of miRNAs observed in circulating levels often correspond to those found in tissues, suggesting miRNA signatures in body fluids follow regular patterns that may reflect underlying physiological or pathological condition [41,42].

### 4.1. MiRNAs in Metabolic Diseases and Obesity

**Figure 5:**
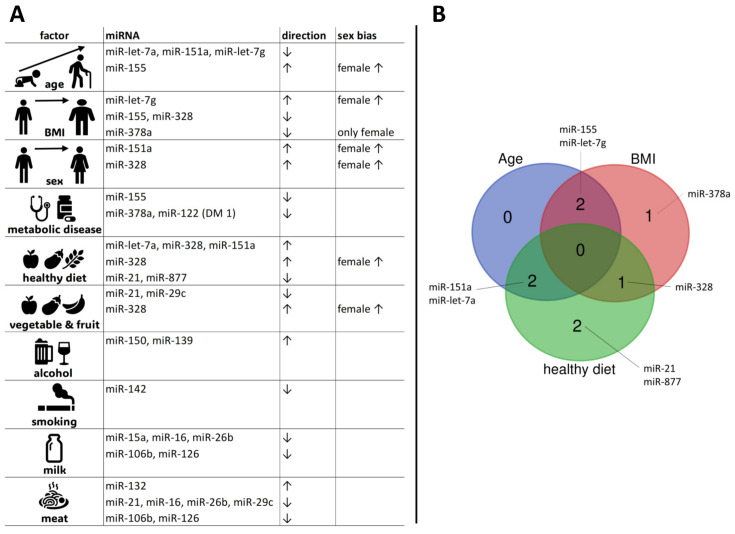
Overview of microRNA expression responses to analysed internal and external factors. Panel (**A**) summarizes the associations between specific miRNAs and the impact of age, body mass index (BMI), sex, metabolic diseases, diet, and vegetable/fruit consumption as well as alcohol intake, smoking, and milk and meat consumption in expression patterns. Arrows indicate the direction of miRNA expression change (↑ for increase, ↓ for decrease) in response to each influence factor. The last column shows whether the factor has a different effect specifically in relation to sex. The table is limited to the presentation of results that are statistically significant, with a *p*-value of less than 0.05. Panel (**B**) uses a Venn diagram illustrating the overlap of microRNAs in response to the key influence factors age, BMI, and a healthy diet rich in fruits, vegetables, and whole grains and low in convenience food.

The vast majority of analysed miRNAs in our study showed altered expression patterns associated with obesity (Figure 2A). Significant results were observed in particular for miR-let-7g, miR-155, miR-328, and miR-378a (Figure 5). This observation is in line with our expectations, considering that adipose tissue is involved in a dynamic exchange with miRNAs and serves as both source and target of miRNAs: On the one hand, the adipose tissue itself, especially infiltrated macrophages and T cells, has been shown to be a major source of a range of circulating exosomal miRNAs in the bloodstream [43]. On the other hand, a variety of miRNAs are known to interact with other adipokines and play an important role in adipose tissue biology [38,44,45,46]. In the context of obesity, the accumulation of immune cells in adipose tissue increases significantly. This phenomenon is characterised by a chronic, low-grade inflammatory state accompanied by elevated serum levels of proinflammatory cytokines [47,48,49], contributing to the occurrence of comorbidities (reviewed in [50]). Research indicates that during inflammation of adipose tissue, the expression of miRNAs is disrupted [18,34]. In our study, miRNA-21 emerged as a potential biomarker that showed a marked, although not statistically significant, increase in expression levels in the obese group, emphasising its broad role in adipogenesis and inflammation. This miRNA is known to promote adipogenesis by modulating and neutralising transforming growth factor β (TGFβ1) [51]. Consistent with our observations, current research has consistently shown a strong positive correlation between miRNA-21 levels and BMI in healthy individuals, further emphasising its central role in obesity [52,53].

Interestingly, we discovered a significant correlation between miR-let-7g and obesity. This miRNA is primarily known as a tumour suppressor that influences the expression of various oncogenic pathways, particularly those associated with c-MYC, and is downregulated in various cancers [30,31,54]. However, there is evidence that altered expression of miR-let-7g may play a role in enhancing intramuscular adipogenesis during foetal muscle development, and over-expressing miR-let-7g in cells leads to reduced adipogenic marker expression and fewer adipocytes forming, along with a decrease in inflammatory cytokine expression [55]. This suggests a mechanism whereby downregulation of miR-let-7g contributes to adipogenic processes associated with obesity, potentially even beyond development phases. Conversely, we observed a significant increase in circulating miR-let-7g with obesity, which could possibly act as a counteracting regulatory mechanism against inflammation and further adipocyte forming. To fully understand these dynamics, further research is essential, focusing on a comprehensive analysis of miRNA levels in adipocytes in tandem with cytokine production studies.

A notably intriguing finding in our study was found for miRNA-155 (Figure 2B), which is known for its role in macrophage recruitment to adipose tissue and its links to insulin resistance, inflammation, and immune response [56,57]. MiR-155 not only interacts with other adipokines, but also downregulates resistin, whose levels are elevated in obesity and related disorders [58]. Interestingly, we found a significant decrease in miR-155 expression with higher BMI, and furthermore, a significantly lower expression in the group of participants with metabolic diseases as well as a strong trend in the same direction for individuals with type 2 diabetes mellitus. This adds to the complex and sometimes contradictory findings regarding miR-155 in obesity and diabetes: for instance, Corral-Fernández et al. observed a downregulation of miR-155 in the peripheral blood mononuclear cells (PBMCs) of patients with severe obesity [59]. Another study showed a reduction in miR-155 expression in PBMCs from type 2 diabetes patients compared to healthy individuals [60]. Lower plasma levels of miR-155 were also correlated with diabetic neuropathy, suggesting a role in the pathogenesis of diabetes, potentially impacting insulin resistance and β-cell loss [61]. In contrast, higher levels of miR-155 were found in adipose tissue macrophages (ATM) in subjects with obesity [62]. A recent study could show a higher expression of miR-155 in obese patients with type 2 diabetes mellitus [63]. These insights highlight miR-155’s complex role in metabolic disorders and it is suggested that its upregulation is linked with adipose tissue health and may counter insulin resistance in type 2 diabetes while enhancing insulin sensitivity in adipose tissue and muscle, aiding liver gluconeogenesis and protecting β-cells under stress [57]. However, a crucial factor to consider is the possible influence of medication on miR-expression in the context of obesity-related inflammation. Levels of miR-155 have been shown to be modulated by TNFα via the NF-κB inflammatory pathway in both cells and exosomes [64]. A commonly prescribed treatment for type 2 diabetes mellitus is metformin, which is reported to interact with this pathway, ultimately leading to a downregulation of miR-155 [65]. Similar effects on this signalling pathway are also known from other drugs used to treat metabolic diseases, including anti-inflammatory drugs. A limitation of our study is that we were unable to accurately quantify the specific drugs and dosages taken by the participants with metabolic diseases, which is an important factor that could influence the interpretation of miR-155 expression levels.

We also observed a strong negative trend in the correlation of miR-15a and miR-16 with type 2 diabetes and obesity, which are reported to be downregulated in insulin resistance and T2D, supporting the potential of both miRNAs to serve as biomarkers for T2D and prediabetes [20,66,67]. Various further miRNAs, including miR-328, miR-30e, miR-378a, miR-26b, miR-106, miR-126, miR-877, miR-378a, miR-122, and miR-142, showed notable trends in the context of obesity. However, their underlying mechanisms, especially regarding their levels in circulation, are not yet fully understood. Many of these miRNAs are critical mediators in metabolism, yet their interplay with obesity and diabetes is complex and multifactorial. These miRNAs may contribute to the inflammatory response triggered by adipose tissue in the early stages of disease development; however, there is a need for further research in this regard. However, our study did not capture exhaustive details on the specific treatments and the precise severity of the metabolic conditions, and thus it is important to note that the primary focus was to investigate the general influence of lifestyle factors and diet beyond and across inflammatory processes on miRNA profiles. Furthermore, when analysing obesity-associated miRNA patterns, individuals with additional metabolic diseases were excluded. Future research should aim to incorporate these variables to build upon our initial findings.

### 4.2. MiRNAs and Ageing

Like obesity, ageing is characterized as a state of chronic inflammation, also known as “inflammageing”. This condition is accompanied by a reshaping of the immune system with an increase in pro-inflammatory molecules in blood and tissues, changes in immune cells, and an altered haematopoiesis, amounting to various diseases like metabolic diseases, autoimmunity, neurodegeneration, and cancer [68,69]. A range of miRNAs have been identified as pivotal in the different hallmarks of ageing [70]. Accordingly, our findings also revealed a multitude of age-dependent trends; however, due to individual variations, only a few results reached statistical significance. For instance, we found an trend indicating an increase in the blood levels of both miRNA-21 and miRNA-146a, two miRNAs that play a crucial role in the context of inflammageing by targeting key molecules in the NF-κB and NLRP3 pathways, essential components of the innate immunity’s signalling mechanisms [34]. During ageing, miR-21 levels increase in muscle and satellite cells, particularly when exposed to reactive oxygen species (ROS) and pro-inflammatory cytokines like IL6 and TNFα [71]. Yet we could observe a significant increase in the expression of miRNA-155 with age. In addition to the previously mentioned association with insulin resistance and obesity, miR-155 is one of the most extensively researched immune-related miRNAs in the context of neuroinflammatory events associated with Alzheimer’s disease [72]. It was shown to be upregulated in both serum and MSCs in older subjects and regulates senescence in mesenchymal stem cells via the Cab39/AMPK pathway. Thus, miRNA-155 represents a potential target to rejuvenate ageing mesenchymal stem cells (MSCs), improving their heart-protective effects [33]. Our findings, along with the results of other studies, reinforce our suggestion that this miRNA is not only a potential marker for diseases such as cancer, metabolic syndrome, and heart failure, as well as inflammatory diseases, but could also serve as potential age-specific prognostic biomarker for healthy ageing.

Significant results were also observed for the two analysed miRNAs from the let-7 family. For both miR-let-7a and miR-let-7g, we observed a decrease in expression in older individuals (Figure 1). Research has demonstrated that let-7 plays a pivotal role in modulating oncogenic pathways through multiple mechanisms of inhibition. In addition to the suppression of various oncogenes as already mentioned [73], Wells et al. demonstrated that let-7 inhibits mTOR activation, which is often activated in tumours and crucial for the generation of reactive oxygen species (ROS) [74]. Moreover, it plays a role in maintaining somatic genome integrity by limiting L1 retrotransposition [75]. Thus, studies have frequently found miR-let-7a as well as miR-let-7g to be downregulated in several cancer types [31,54,76,77] and to also promote anti-inflammatory factors [78]. We found no data in the literature on circulating levels of miR-let-7a in the blood of healthy ageing individuals. However, we hypothesise that the reduced expression of both miR-let-7a and miR-let-7g we observed may contribute to increased susceptibility to age-related diseases such as cancer in ageing individuals. This could be due a reduced immune function for example via altered T cell activation as well as genomic instability and increased oxidative stress.

This distinctive set of circulating miRNAs, altered in ageing, may not only serve as indicators for biological age but also could act as biomarkers for assessing the risk of inflammation-related age conditions.

### 4.3. Impact of Lifestyle Choices: Smoking, Alcohol, and miRNA Expression

Lifestyle choices have been increasingly recognized for their impact on the regulation of miRNAs. For instance, several studies have attempted to identify characteristic miRNA profiles in plasma that are associated with smoking, with sometimes divergent and sometimes overlapping results [26,79,80]. The key miRNAs identified by Willinger et al., including miRNA-181a, were additionally associated with systemic inflammatory markers and reduced lung function [26]. They also correlated with genes involved in immune function, supporting the hypothesis that smoking-associated miRNAs promote smoking-induced inflammation. However, we did not find any significant effects on this miRNA. Interestingly, we found a notable correlation with miRNA-142 (Figure 3), whose downregulation has been linked to lung cancer in recent studies, for instance via the CYR61-Wnt/β-catenin axis [81,82,83]. For future research, it would be interesting to correlate oxidative stress markers, typically elevated in smokers, with specific miRNAs.

Like tobacco smoke, alcohol can interact with cellular processes, potentially leading to changes in miRNA profiles, ultimately resulting in a range of diseases. However, it is important to point out that there were no known cases of alcoholism in our study group and the subgroup of frequent alcohol drinkers was quite small. Furthermore, we did not quantify the exact amounts of alcohol consumed but assessed the frequency of drinking. Consequently, we found significant results only for the group of moderate alcohol consumers. In this group, miRNA-150 and miR-139 were significantly elevated compared to non-drinkers (Figure 3). MiR-150 is suggested to play a protective role in the heart [84], while MiR-139 is discussed as gate-keeper in cancer while acting against different types of stress, including DNA damage [85]. This may indicate a specific regulatory pathway through which moderate alcohol consumption exerts its protective effects on heart health.

### 4.4. Diet and Nutrimiromics

The transition from nutrigenetics to the so called ‘nutrimiromics’ marks a significant deepening of our understanding of the complex interaction among gene expression, miRNAs, and dietary influences, ultimately affecting susceptibility to diseases [86]. To explore the correlation between a healthy dietary pattern and miRNA expression, we categorized the participants into two clusters based on their dietary habits, taking into account potential confounders such as physical activity and BMI. Within the cohort consuming a diet rich in vegetables, fruits, and whole grains, and low in processed convenience foods, we observed an elevated expression of miR-let-7a and miR-328 along with a decreased expression of miR-21. As previously mentioned, miR-21 serves both as pro-inflammatory and oncogenic miRNA, playing a pivotal role in inflammageing. Notably, our results indicated a significant reduction in miR-21 levels not only in response to a generally healthy diet but also, more specifically, upon analysing the consumption of fruits and vegetables. A healthy dietary pattern has the potential to modulate miRNA activity through various pathways: bioactive compounds such as resveratrol, curcumin, quercetin, and catechins as well as minerals or vitamins and polyunsaturated fatty acids (PUFAS) have been reported to directly modulate miRNA expression [86,87,88,89,90,91,92]. For instance, eicosapentaenoic (EPA) and docosahexaenoic (DHA) acids as well as polyphenols exert part of their anti-inflammatory action by downregulating miRNAs like miR-146a, miR-21, and miR-155, particularly after macrophage and epithelial cell stimulation while mediating NF-κB signalling [64,92]. Conversely, high-fat diets rich in saturated fats like lauric and palmitic acids can trigger inflammatory pathways through stimulation of pro-inflammatory miRNAs such as miR-155 [93]. This suggests that higher intake of foods rich in these compounds may directly or indirectly mediate protection against inflammatory processes and related comorbidities. This aligns well with our findings on miR-let-7a, which, as described above, exerts various protective mechanisms against a range of diseases. The specific mechanisms through which diet enhances miR-let-7a expression are yet to be fully determined. Anticipated pathways involve changes in gene expression induced by micronutrients via transcription factors and/or epigenetic modifications, such as DNA methylation, as well as interactions with other non-coding RNAs. Additionally, the role of the gut microbiome in generating short-chain fatty acids (SCFAs) from dietary fibre, potentially influencing miRNA activity, is another critical area for further exploration. These outlined mechanisms could similarly account for the noted elevation in miRNA-328 levels; however, the effects of the observed increase in miRNA-328 expression remain unclear given its multifaceted role in various tumour types. While it acts as a tumour suppressor in various cancers by inhibiting tumour progression, miRNA-328 paradoxically acts as an onco-miRNA in ovarian cancer, highlighting the complexity of its function in oncogenesis and the need for further research to decipher its diverse effects [94,95,96,97,98].

Recently, however, another significant aspect has emerged regarding the interplay between diet and miRNAs: food-derived miRNAs. Yet they are a complex topic with different conclusions about their impact and bioavailability. While research has shown that food-derived miRNAs from plants like rice can be absorbed into mammalian plasma and tissues, other studies, like those involving intake of vegetables, show no significant change in miRNA levels in human plasma [99,100]. Recently, the focus has shifted to miRNAs in animal-based foods, particularly cow’s milk. It is now understood that exogenous miRNAs from milk can enter the human body and potentially regulate human metabolism by affecting gene expression. Milk-derived miRNAs, likely more resistant to enzymatic digestion due to their presence in extracellular vesicles, suggest a role in cross-organism and cell to cell communication [101,102,103]. However, food processing methods like microwaving, yogurt fermentation, and cheese production can significantly decrease the levels of these miRNAs [104]. We could identify several miRNAs differently expressed in subjects with higher milk consumption, including miR-15a, miR-16, miR-26b, miR-106b, and miR-126. The miR-15/16 cluster, as well as miR-126, is reported to act as tumour suppressors, while miR-106b was upregulated in cancer [32,105]. Interestingly, an association between miRNA-106b and dairy products was found in a previous study [106]. In our case, where lower levels of circulating miRNAs were observed, we hypothesise that the effects are not directly due to dietary miRNAs in milk, but rather to milk minerals or vitamins such as zinc, calcium, or vitamin D. These components are closely associated with the modulation of various miRNAs, particularly in the context of inhibition of cancer initiation and development [88,107,108,109]. However, milk has also been repeatedly associated with an increased risk of certain cancers, such as prostate cancer, where the insulin-like growth factor (IGF) signalling pathway, which is known to interact with miRNAs, may be stimulated by milk. Our results reflect that the effects of milk consumption may be multifaceted, with potentially both beneficial and detrimental influences. Regarding meat consumption, our research has also uncovered varied and distinct outcomes. Our findings showed that daily meat consumers had notably higher miR-132 expression but lower levels of miR-21, miR-16, miR-26b, miR-29c, miR-106b, and miR-126. There are few studies on the possible relationship between meat consumption and circulating miRNAs. However, it has been observed that a diet high in red meat can alter miRNA levels in rectal mucosal tissue, upregulating oncomirs such as miR-21. MiR-132, upregulated in our study in high meat consumers, is associated with neuroinflammation and is involved in signalling pathways related to the pathogenesis of acute myocardial infarction [110,111]. Although meat can provide important nutrients and high-quality protein, there is a fundamental difference between the types of meat consumed, which was not assessed in this study. Future research should focus more on the type of meat (red or white) and its processing to investigate these aspects. Our results, combined with recent research findings, show that a healthy diet is closely linked to miRNAs potentially exert an inhibition of inflammatory processes and resulting comorbidities.

### 4.5. Gender-Specific Differences, Immune System, and Personalized Interventions

Sex as a biological variable has been increasingly recognized as a crucial element in research design and analysis over the past decade [6,112,113,114]. Numerous studies have highlighted the sex-biased susceptibility to diseases, with the interaction of sex chromosomes and hormones often cited as a primary cause [7,115,116]. The regulation of miRNA expression from a sex perspective is particularly interesting. It has been noted that this regulation is primarily influenced by two factors: the presence of miRNAs on the X chromosome and the modulation of miRNA transcription and processing by oestrogen [117]. Recent research has revealed that miRNAs are not only expressed in a sex-specific manner but also play a pivotal role in driving sex-specific immune responses, contributing to the different health outcomes observed in men and women. For instance, miRNAs located on the X chromosome have been implicated in the sex-biased development of immune-mediated diseases [118]. Cui et al. discovered 73 female-biased and 163 male-biased miRNAs [119]. In our study, we observed a sex-specific expression of most of the analysed miRNAs; however, across the entire group, only miR-151a showed a significant positive bias towards females (Figure 4). The gene encoding this miRNA is located on chromosome 8, which precludes the possibility of sex bias arising from X-chromosome inactivation escape. It is well-established that sex hormones such as oestradiol, progesterone, and testosterone have the potential to modulate miRNA expression, impacting both directly and indirectly on miRNA processing pathways. The hormonal regulation of miR-151a, however, remains an area for further exploration. This notwithstanding, evidence suggests that miR-151a expression is sex-dependent, with possible hormonal influences. For instance, studies on polycystic ovary syndrome (PCOS) indicate that variations in exosomal miRNA levels, including miR-151a, correlate with hormonal fluctuations, hinting at a relationship between hormone profiles and miRNA expression [120]. A recent study dated January 2024 further delineates gender-specific differences in miR-151 expression in mice, observing suppression in WT females subjected to tobacco smoke [121]. Our analysis revealed no significant association between smoking and the expression of this specific miRNA. However, the data did indicate a trend towards a more pronounced negative difference in expression between smokers and non-smokers among females compared to males, with mean differences of −0.145 for females and −0.45 for males. This points to the role of environmental factors, alongside hormonal influences, in modulating miR-151a expression, underscoring the nuanced regulation of this miR. A notable positive association was observed between age and the expression of miR-155 in females. MiR-155, as described above, is known to play a crucial role in both innate and adaptive immunity. Its abnormal expression is associated with various inflammatory autoimmune diseases such as rheumatoid arthritis, systemic lupus erythematosus, and multiple sclerosis [22]. Furthermore, a study indicated sex-specific disparities in miR-155 expression in innate lymphoid cells (ILCs). ILCs, which are potent drivers of inflammation and the innate counterparts of T helper cells, exhibited higher miR-155 expression levels in female-derived cells in response to various stimuli [122].

When analysing possible differential effects of different lifestyle aspects on gender, we could observe variances in miR-378a in the context of obesity and miR-328 in the context of a healthy diet in females. This observation underscores the importance of tailoring lifestyle and dietary interventions not only to individual health status but also to gender. Understanding sex-specific gene expression is central to precision medicine as it helps in the development of customised strategies for disease treatment and prevention. The various molecular patterns associated with sex differences are of critical importance, especially since female miRNAs are frequently associated with several diseases, including cancer and neurodegenerative diseases. Recognising these differences may improve our overall understanding of the role of biological sex in health outcomes.

## 5. Conclusions

Our study once again highlights the intricate relationship between lifestyle factors, individual health status, gender, and the fine-tuning of metabolic processes by miRNAs, underlining their central role in personalised prevention strategies. MiRNAs serve as dynamic biomarkers that reflect unique biological and environmental signatures and pave the way for customised and effective medical interventions. Our focus on circulating miRNAs leverages their accessibility from body fluids for non-invasive health monitoring that aligns with the goals of personalised medicine. In acknowledging gender differences, we emphasize the importance of considering biological sex in the analysis of miRNA expression patterns, which can influence inflammation and immune responses, thereby impacting systemic disease. While our study did not standardize sampling times, this aspect is particularly interesting for future research given the emerging understanding that circulating miRNAs may exhibit diurnal variations, the extent of which remains to be fully elucidated. In the future, the use of artificial intelligence to decipher the vast amounts of data and complex metabolic networks could help drive personalised nutritional advice and optimise health outcomes through tailored dietary recommendations.

## Figures and Tables

**Table 1:** Demographic and health characteristics of the study participants.

Study Population	*N*	Age ± SD (Years)	Age Range (Years)	BMI ± SD (kg/m^2^)	BMI Range (kg/m^2^)	Female (*N*)	Male (*N*)
Healthy	197	49.17 ± 13.18	21–80	23.67 ± 3.02	17.72–29.90	130	67
Metabolic disease	108	53.59 ± 13.78	20–92	28.20 ± 6.14	16.37–45.55	79	29
Thyroid dysfunction	48	54.21 ± 13.68	20–92	24.60 ± 4.34	16.37–37.13	44	4
Obesity	37	50.19 ± 13.65	23–76	33.59 ± 3.76	30.08–45.55	25	12
Type 2 diabetes mellitus	7	58.29 ± 17.54	24–74	30.24 ± 6.54	24.74–43.51	3	4
Type 1 diabetes mellitus	4	51.25 ± 16.34	35–69	28.14 ± 6.59	21.87–37.18	1	3
Gout	4	58.75 ± 16.28	47–82	22.98 ± 5.84	18.56–30.86	2	2
Other	4	61.00 ± 2.45	58–64	27.15 ± 7.53	21.63–38.22	2	2
Lipid metabolism dysfunction	3	50.67 ± 12.22	40–64	23.06 ± 4.30	19.36–27.77	2	1
Glucose intolerance	1	55	20–92	28.29		44	1
Whole sample group	305	50.74 ± 13.54	20–92	25.27 ± 4.88	16.37–45.55	209	96

**Table 2:** Means, adjusted means, standard deviations (SD) and standard errors (SE) for expression levels of miR-142 for three smoking behaviours.

Smoking Behaviour									
	Whole Sample Group			Healthy			Metabolic Disease		
delta CT miR-142	no	former	daily	no	former	daily	no	former	daily
M	3.009	2.857	3.446	3.010	2.603	3.397	3.006	3.131	3.535
(SD)	(0.678)	(0.865)	(0.771)	(0.723)	(0.860)	(0.631)	(0.581)	(0.814)	(1.059)
M_adj_	2.998	2.818	3.546	2.882	2.390	3.510	2.925	3.154	3.775
(SE)	(0.153)	(0.154)	(0.215)	(0.263)	(0.222)	(0.275)	(0.276)	(0.296)	(0.472)

Note. MicroRNA expression was quantified as delta CT values, where CT = threshold cycle, delta CT = (CT target miRNA minus CT miRNA 93 + 24).

## Data Availability

Data are contained within the article.

## References

[1] Asghar W., Khalid N. (2023). Nutrigenetics and Nutrigenomics, and Precision Nutrition. Nutr. Health.

[2] Ramos-Lopez O., Martinez J.A., Milagro F.I. (2022). Holistic Integration of Omics Tools for Precision Nutrition in Health and Disease. Nutrients.

[3] Missong H., Joshi R., Khullar N., Thareja S., Navik U., Bhatti G.K., Bhatti J.S. (2024). Nutrient-Epigenome Interactions: Implications for Personalized Nutrition against Aging-Associated Diseases. J. Nutr. Biochem..

[4] Berciano S., Figueiredo J., Brisbois T.D., Alford S., Koecher K., Eckhouse S., Ciati R., Kussmann M., Ordovas J.M., Stebbins K. (2022). Precision Nutrition: Maintaining Scientific Integrity While Realizing Market Potential. Front. Nutr..

[5] Manuel R.S.J., Liang Y. (2021). Sexual Dimorphism in Immunometabolism and Autoimmunity: Impact on Personalized Medicine. Autoimmun. Rev..

[6] Prajapati C., Koivumäki J., Pekkanen-Mattila M., Aalto-Setälä K. (2022). Sex Differences in Heart: From Basics to Clinics. Eur. J. Med. Res..

[7] Lipoldová M., Demant P. (2021). Gene-Specific Sex Effects on Susceptibility to Infectious Diseases. Front. Immunol..

[8] Brennan L., de Roos B. (2021). Nutrigenomics: Lessons Learned and Future Perspectives. Am. J. Clin. Nutr..

[9] Abdellaoui A., Yengo L., Verweij K.J.H., Visscher P.M. (2023). 15 Years of GWAS Discovery: Realizing the Promise. Am. J. Hum. Genet..

[10] Sud A., Kinnersley B., Houlston R.S. (2017). Genome-Wide Association Studies of Cancer: Current Insights and Future Perspectives. Nat. Rev. Cancer.

[11] Oo J.A., Brandes R.P., Leisegang M.S. (2022). Long Non-Coding RNAs: Novel Regulators of Cellular Physiology and Function. Pflugers Arch..

[12] Quinodoz S., Guttman M. (2014). Long Noncoding RNAs: An Emerging Link between Gene Regulation and Nuclear Organization. Trends Cell Biol..

[13] Gregory R.I., Chendrimada T.P., Cooch N., Shiekhattar R. (2005). Human RISC Couples MicroRNA Biogenesis and Posttranscriptional Gene Silencing. Cell.

[14] Elshazli R.M., Toraih E.A., Hussein M.H., Ruiz E.M., Kandil E., Fawzy M.S. (2023). Pan-Cancer Study on Variants of Canonical miRNA Biogenesis Pathway Components: A Pooled Analysis. Cancers.

[15] Liu W., Wang X. (2019). Prediction of Functional microRNA Targets by Integrative Modeling of microRNA Binding and Target Expression Data. Genome Biol..

[16] Raisch J., Darfeuille-Michaud A., Nguyen H.T.T. (2013). Role of microRNAs in the Immune System, Inflammation and Cancer. World J. Gastroenterol..

[17] Zhou H., Ni W.-J., Meng X.-M., Tang L.-Q. (2021). MicroRNAs as Regulators of Immune and Inflammatory Responses: Potential Therapeutic Targets in Diabetic Nephropathy. Front. Cell Dev. Biol..

[18] Oses M., Margareto Sanchez J., Portillo M.P., Aguilera C.M., Labayen I. (2019). Circulating miRNAs as Biomarkers of Obesity and Obesity-Associated Comorbidities in Children and Adolescents: A Systematic Review. Nutrients.

[19] Roy B., Lee E., Li T., Rampersaud M. (2022). Role of miRNAs in Neurodegeneration: From Disease Cause to Tools of Biomarker Discovery and Therapeutics. Genes.

[20] Mastropasqua R., D’Aloisio R., Costantini E., Porreca A., Ferro G., Libertini D., Reale M., Di Nicola M., Viggiano P., Falconio G. (2021). Serum microRNA Levels in Diabetes Mellitus. Diagnostics.

[21] Li X., Dai A., Tran R., Wang J. (2023). Identifying miRNA Biomarkers for Breast Cancer and Ovarian Cancer: A Text Mining Perspective. Breast Cancer Res. Treat..

[22] Xu W.-D., Feng S.-Y., Huang A.-F. (2022). Role of miR-155 in Inflammatory Autoimmune Diseases: A Comprehensive Review. Inflamm. Res..

[23] Krammer U.D.B., Tschida S., Berner J., Lilja S., Switzeny O.J., Hippe B., Rust P., Haslberger A.G. (2022). MiRNA-Based “Fitness Score” to Assess the Individual Response to Diet, Metabolism, and Exercise. J. Int. Soc. Sports Nutr..

[24] Vrijens K., Bollati V., Nawrot T.S. (2015). MicroRNAs as Potential Signatures of Environmental Exposure or Effect: A Systematic Review. Environ. Health Perspect..

[25] Zhou Q., Shi C., Lv Y., Zhao C., Jiao Z., Wang T. (2020). Circulating microRNAs in Response to Exercise Training in Healthy Adults. Front. Genet..

[26] Willinger C.M., Rong J., Tanriverdi K., Courchesne P.L., Huan T., Wasserman G.A., Lin H., Dupuis J., Joehanes R., Jones M.R. (2017). A MicroRNA Signature of Cigarette Smoking and Evidence for a Putative Causal Role of MicroRNAs in Smoking-Related Inflammation and Target Organ Damage. Circ. Cardiovasc. Genet..

[27] Kura B., Parikh M., Slezak J., Pierce G.N. (2019). The Influence of Diet on MicroRNAs That Impact Cardiovascular Disease. Molecules.

[28] Wang H., Peng R., Wang J., Qin Z., Xue L. (2018). Circulating microRNAs as Potential Cancer Biomarkers: The Advantage and Disadvantage. Clin. Epigenet..

[29] Mitchell P.S., Parkin R.K., Kroh E.M., Fritz B.R., Wyman S.K., Pogosova-Agadjanyan E.L., Peterson A., Noteboom J., O’Briant K.C., Allen A. (2008). Circulating microRNAs as Stable Blood-Based Markers for Cancer Detection. Proc. Natl. Acad. Sci. USA.

[30] Chirshev E., Oberg K.C., Ioffe Y.J., Unternaehrer J.J. (2019). Let-7 as Biomarker, Prognostic Indicator, and Therapy for Precision Medicine in Cancer. Clin. Transl. Med..

[31] Biamonte F., Santamaria G., Sacco A., Perrone F.M., Di Cello A., Battaglia A.M., Salatino A., Di Vito A., Aversa I., Venturella R. (2019). MicroRNA Let-7g Acts as Tumor Suppressor and Predictive Biomarker for Chemoresistance in Human Epithelial Ovarian Cancer. Sci. Rep..

[32] Aqeilan R.I., Calin G.A., Croce C.M. (2010). miR-15a and miR-16-1 in Cancer: Discovery, Function and Future Perspectives. Cell Death Differ..

[33] Hong Y., He H., Jiang G., Zhang H., Tao W., Ding Y., Yuan D., Liu J., Fan H., Lin F. (2020). miR-155-5p Inhibition Rejuvenates Aged Mesenchymal Stem Cells and Enhances Cardioprotection Following Infarction. Aging Cell.

[34] Olivieri F., Prattichizzo F., Giuliani A., Matacchione G., Rippo M.R., Sabbatinelli J., Bonafè M. (2021). miR-21 and miR-146a: The microRNAs of Inflammaging and Age-Related Diseases. Ageing Res. Rev..

[35] Huang X.-Y., Chen J.-X., Ren Y., Fan L.-C., Xiang W., He X.-J. (2022). Exosomal miR-122 Promotes Adipogenesis and Aggravates Obesity through the VDR/SREBF1 Axis. Obesity.

[36] Zhang X., Dong S., Jia Q., Zhang A., Li Y., Zhu Y., Lv S., Zhang J. (2019). The microRNA in Ventricular Remodeling: The miR-30 Family. Biosci. Rep..

[37] Thermo Fisher Scientific (2016). A Technical Guide to Identifying miRNA Normalizers Using TaqMan Advanced miRNA Assays.

[38] Iacomino G., Siani A. (2017). Role of microRNAs in Obesity and Obesity-Related Diseases. Genes Nutr..

[39] Loganathan T., Doss C.G.P. (2023). Non-Coding RNAs in Human Health and Disease: Potential Function as Biomarkers and Therapeutic Targets. Funct. Integr. Genom..

[40] He B., Zhao Z., Cai Q., Zhang Y., Zhang P., Shi S., Xie H., Peng X., Yin W., Tao Y. (2020). miRNA-Based Biomarkers, Therapies, and Resistance in Cancer. Int. J. Biol. Sci..

[41] Cui C., Cui Q. (2020). The Relationship of Human Tissue microRNAs with Those from Body Fluids. Sci. Rep..

[42] Waters P.S., McDermott A.M., Wall D., Heneghan H.M., Miller N., Newell J., Kerin M.J., Dwyer R.M. (2012). Relationship between Circulating and Tissue microRNAs in a Murine Model of Breast Cancer. PLoS ONE.

[43] Thomou T., Mori M.A., Dreyfuss J.M., Konishi M., Sakaguchi M., Wolfrum C., Rao T.N., Winnay J.N., Garcia-Martin R., Grinspoon S.K. (2017). Adipose-Derived Circulating miRNAs Regulate Gene Expression in Other Tissues. Nature.

[44] Rakib A., Kiran S., Mandal M., Singh U.P. (2022). MicroRNAs: A Crossroad That Connects Obesity to Immunity and Aging. Immun. Ageing.

[45] Silveira A., Gomes J., Roque F., Fernandes T., de Oliveira E.M. (2022). MicroRNAs in Obesity-Associated Disorders: The Role of Exercise Training. Obes. Facts.

[46] Deiuliis J.A. (2016). MicroRNAs as Regulators of Metabolic Disease: Pathophysiologic Significance and Emerging Role as Biomarkers and Therapeutics. Int. J. Obes..

[47] Bulló M., García-Lorda P., Megias I., Salas-Salvadó J. (2003). Systemic Inflammation, Adipose Tissue Tumor Necrosis Factor, and Leptin Expression. Obes. Res..

[48] Mabrouk R., Ghareeb H., Shehab A., Omar K., El-Kabarity R.H., Soliman D.A., Mohamed N.A. (2013). Serum Visfatin, Resistin and IL-18 in A Group of Egyptian Obese Diabetic and Non Diabetic Individuals. Egypt. J. Immunol..

[49] Festa A., D’Agostino R., Williams K., Karter A.J., Mayer-Davis E.J., Tracy R.P., Haffner S.M. (2001). The Relation of Body Fat Mass and Distribution to Markers of Chronic Inflammation. Int. J. Obes. Relat. Metab. Disord. J. Int. Assoc. Study Obes..

[50] Clemente-Suárez V.J., Redondo-Flórez L., Beltrán-Velasco A.I., Martín-Rodríguez A., Martínez-Guardado I., Navarro-Jiménez E., Laborde-Cárdenas C.C., Tornero-Aguilera J.F. (2023). The Role of Adipokines in Health and Disease. Biomedicines.

[51] Kim Y.J., Hwang S.J., Bae Y.C., Jung J.S. (2009). MiR-21 Regulates Adipogenic Differentiation through the Modulation of TGF-Beta Signaling in Mesenchymal Stem Cells Derived from Human Adipose Tissue. Stem Cells.

[52] Keller P., Gburcik V., Petrovic N., Gallagher I.J., Nedergaard J., Cannon B., Timmons J.A. (2011). Gene-Chip Studies of Adipogenesis-Regulated microRNAs in Mouse Primary Adipocytes and Human Obesity. BMC Endocr. Disord..

[53] Lhamyani S., Gentile A.-M., Giráldez-Pérez R.M., Feijóo-Cuaresma M., Romero-Zerbo S.Y., Clemente-Postigo M., Zayed H., Oliva-Olivera W., Bermúdez-Silva F.J., Salas J. (2021). miR-21 Mimic Blocks Obesity in Mice: A Novel Therapeutic Option. Mol. Ther.—Nucleic Acids.

[54] Yazarlou F., Kadkhoda S., Ghafouri-Fard S. (2021). Emerging Role of Let-7 Family in the Pathogenesis of Hematological Malignancies. Biomed. Pharmacother..

[55] Yan X., Huang Y., Zhao J.-X., Rogers C.J., Zhu M.-J., Ford S.P., Nathanielsz P.W., Du M. (2013). Maternal Obesity Downregulates microRNA Let-7g Expression, a Possible Mechanism for Enhanced Adipogenesis during Ovine Fetal Skeletal Muscle Development. Int. J. Obes..

[56] Karkeni E., Astier J., Tourniaire F., El Abed M., Romier B., Gouranton E., Wan L., Borel P., Salles J., Walrand S. (2016). Obesity-Associated Inflammation Induces microRNA-155 Expression in Adipocytes and Adipose Tissue: Outcome on Adipocyte Function. J. Clin. Endocrinol. Metab..

[57] Jankauskas S.S., Gambardella J., Sardu C., Lombardi A., Santulli G. (2021). Functional Role of miR-155 in the Pathogenesis of Diabetes Mellitus and Its Complications. Non-Coding RNA.

[58] Johnson C., Drummer C., Virtue A., Gao T., Wu S., Hernandez M., Singh L., Wang H., Yang X.-F. (2018). Increased Expression of Resistin in MicroRNA-155-Deficient White Adipose Tissues May Be a Possible Driver of Metabolically Healthy Obesity Transition to Classical Obesity. Front. Physiol..

[59] Corral-Fernández N.E., Salgado-Bustamante M., Martínez-Leija M.E., Cortez-Espinosa N., García-Hernández M.H., Reynaga-Hernández E., Quezada-Calvillo R., Portales-Pérez D.P. (2013). Dysregulated miR-155 Expression in Peripheral Blood Mononuclear Cells from Patients with Type 2 Diabetes. Exp. Clin. Endocrinol. Diabetes.

[60] Mazloom H., Alizadeh S., Pasalar P., Esfahani E.N., Meshkani R. (2015). Downregulated microRNA-155 Expression in Peripheral Blood Mononuclear Cells of Type 2 Diabetic Patients Is Not Correlated with Increased Inflammatory Cytokine Production. Cytokine.

[61] Ciccacci C., Latini A., Colantuono A., Politi C., D’Amato C., Greco C., Rinaldi M.E., Lauro D., Novelli G., Spallone V. (2020). Expression Study of Candidate miRNAs and Evaluation of Their Potential Use as Biomarkers of Diabetic Neuropathy. Epigenomics.

[62] Tryggestad J.B., Teague A.M., Sparling D.P., Jiang S., Chernausek S.D. (2019). Macrophage Derived MicroRNA-155 Increases in Obesity and Influences Adipocyte Metabolism by Targeting Peroxisome Proliferator-Activated Receptor Gamma. Obesity.

[63] Catanzaro G., Conte F., Trocchianesi S., Splendiani E., Bimonte V.M., Mocini E., Filardi T., Po A., Besharat Z.M., Gentile M.C. (2023). Network Analysis Identifies Circulating miR-155 as Predictive Biomarker of Type 2 Diabetes Mellitus Development in Obese Patients: A Pilot Study. Sci. Rep..

[64] Merve Bayram H., Eren F., Esra Gunes F. (2021). The Relationship between Polyphenols and miRNAs: A Novel Therapeutic Strategy for Metabolic Associated Fatty Liver Disease. Hepatol. Forum.

[65] Kanigur Sultuybek G., Soydas T., Yenmis G. (2019). NF-κB as the Mediator of Metformin’s Effect on Ageing and Ageing-Related Diseases. Clin. Exp. Pharmacol. Physiol..

[66] Al-Kafaji G., Al-Mahroos G., Alsayed N.A., Hasan Z.A., Nawaz S., Bakhiet M. (2015). Peripheral Blood microRNA-15a Is a Potential Biomarker for Type 2 Diabetes Mellitus and Pre-Diabetes. Mol. Med. Rep..

[67] Lee D.E., Brown J.L., Rosa M.E., Brown L.A., Perry R.A., Wiggs M.P., Nilsson M.I., Crouse S.F., Fluckey J.D., Washington T.A. (2016). microRNA-16 Is Downregulated During Insulin Resistance and Controls Skeletal Muscle Protein Accretion. J. Cell. Biochem..

[68] Ferrucci L., Fabbri E. (2018). Inflammageing: Chronic Inflammation in Ageing, Cardiovascular Disease, and Frailty. Nat. Rev. Cardiol..

[69] Montecino-Rodriguez E., Berent-Maoz B., Dorshkind K. (2013). Causes, Consequences, and Reversal of Immune System Aging. J. Clin. Investig..

[70] Harries L.W. (2014). MicroRNAs as Mediators of the Ageing Process. Genes.

[71] Borja-Gonzalez M., Casas-Martinez J.C., McDonagh B., Goljanek-Whysall K. (2020). Inflamma-miR-21 Negatively Regulates Myogenesis during Ageing. Antioxidants.

[72] Guedes J.R., Custódia C.M., Silva R.J., de Almeida L.P., Pedroso de Lima M.C., Cardoso A.L. (2014). Early miR-155 Upregulation Contributes to Neuroinflammation in Alzheimer’s Disease Triple Transgenic Mouse Model. Hum. Mol. Genet..

[73] Ma Y., Shen N., Wicha M.S., Luo M. (2021). The Roles of the Let-7 Family of MicroRNAs in the Regulation of Cancer Stemness. Cells.

[74] Wells A.C., Hioki K.A., Angelou C.C., Lynch A.C., Liang X., Ryan D.J., Thesmar I., Zhanybekova S., Zuklys S., Ullom J. (2023). Let-7 Enhances Murine Anti-Tumor CD8 T Cell Responses by Promoting Memory and Antagonizing Terminal Differentiation. Nat. Commun..

[75] Tristán-Ramos P., Rubio-Roldan A., Peris G., Sánchez L., Amador-Cubero S., Viollet S., Cristofari G., Heras S.R. (2020). The Tumor Suppressor microRNA Let-7 Inhibits Human LINE-1 Retrotransposition. Nat. Commun..

[76] Pulliero A., Mastracci L., Tarantini L., Khalid Z., Bollati V., Izzotti A. (2023). Let-7a Downregulation Accompanied by KRAS Mutation Is Predictive of Lung Cancer Onset in Cigarette Smoke-Exposed Mice. Int. J. Mol. Sci..

[77] Asghari F., Haghnavaz N., Shanehbandi D., Khaze V., Baradaran B., Kazemi T. (2018). Differential Altered Expression of Let-7a and miR-205 Tumor-Suppressor miRNAs in Different Subtypes of Breast Cancer under Treatment with Taxol. Adv. Clin. Exp. Med..

[78] Cho K.J., Song J., Oh Y., Lee J.E. (2015). MicroRNA-Let-7a Regulates the Function of Microglia in Inflammation. Mol. Cell. Neurosci..

[79] Takahashi K., Yokota S., Tatsumi N., Fukami T., Yokoi T., Nakajima M. (2013). Cigarette Smoking Substantially Alters Plasma microRNA Profiles in Healthy Subjects. Toxicol. Appl. Pharmacol..

[80] Karabegović I., Maas S.C.E., Shuai Y., Ikram M.A., Stricker B., Aerts J., Brusselle G., Lahousse L., Voortman T., Ghanbari M. (2023). Smoking-Related Dysregulation of Plasma Circulating microRNAs: The Rotterdam Study. Hum. Genom..

[81] Wu X.-B., Li Q.-H., Zhang N., Li M., Li K. (2019). MiR-142 Inhibits Lung Cancer Cell Proliferation and Promotes Apoptosis by Targeting XIAP. Eur. Rev. Med. Pharmacol. Sci..

[82] Yan J., Yang B., Lin S., Xing R., Lu Y. (2019). Downregulation of miR-142-5p Promotes Tumor Metastasis through Directly Regulating CYR61 Expression in Gastric Cancer. Gastric Cancer Off. J. Int. Gastric Cancer Assoc. Jpn. Gastric Cancer Assoc..

[83] Yang T., He F., Zhang M., Ai L., He M., Liu X., Li Y. (2022). MiR-142-3p as an Indicator of OSA Severity Predicts Prognosis in Lung Adenocarcinoma with OSA. Nat. Sci. Sleep.

[84] Aonuma T., Moukette B., Kawaguchi S., Barupala N.P., Sepúlveda M.N., Corr C., Tang Y., Liangpunsakul S., Payne R.M., Willis M.S. (2021). Cardiomyocyte microRNA-150 Confers Cardiac Protection and Directly Represses Proapoptotic Small Proline-Rich Protein 1A. JCI Insight.

[85] Stavast C.J., van Zuijen I., Erkeland S.J. (2022). MicroRNA-139, an Emerging Gate-Keeper in Various Types of Cancer. Cells.

[86] Quintanilha B.J., Reis B.Z., Duarte G.B.S., Cozzolino S.M.F., Rogero M.M. (2017). Nutrimiromics: Role of microRNAs and Nutrition in Modulating Inflammation and Chronic Diseases. Nutrients.

[87] Li H., Jia Z., Li A., Jenkins G., Yang X., Hu J., Guo W. (2013). Resveratrol Repressed Viability of U251 Cells by miR-21 Inhibiting of NF-κB Pathway. Mol. Cell. Biochem..

[88] Zhang Z., Moon R., Thorne J.L., Moore J.B. (2023). NAFLD and Vitamin D: Evidence for Intersection of microRNA-Regulated Pathways. Nutr. Res. Rev..

[89] Huang X., Dong Y.-L., Li T., Xiong W., Zhang X., Wang P.-J., Huang J.-Q. (2021). Dietary Selenium Regulates microRNAs in Metabolic Disease: Recent Progress. Nutrients.

[90] Ibrahim S.S.A., Kandil L.S., Ragab G.M., El-Sayyad S.M. (2021). Micro RNAs 26b, 20a Inversely Correlate with GSK-3 β/NF-κB/NLRP-3 Pathway to Highlight the Additive Promising Effects of Atorvastatin and Quercetin in Experimental Induced Arthritis. Int. Immunopharmacol..

[91] Majidinia M., Karimian A., Alemi F., Yousefi B., Safa A. (2020). Targeting miRNAs by Polyphenols: Novel Therapeutic Strategy for Aging. Biochem. Pharmacol..

[92] Roessler C., Kuhlmann K., Hellwing C., Leimert A., Schumann J. (2017). Impact of Polyunsaturated Fatty Acids on miRNA Profiles of Monocytes/Macrophages and Endothelial Cells—A Pilot Study. Int. J. Mol. Sci..

[93] Takahashi K., Sasano T., Sugiyama K., Kurokawa J., Tamura N., Soejima Y., Sawabe M., Isobe M., Furukawa T. (2016). High-Fat Diet Increases Vulnerability to Atrial Arrhythmia by Conduction Disturbance via miR-27b. J. Mol. Cell. Cardiol..

[94] Iwańczyk S., Lehmann T., Cieślewicz A., Malesza K., Woźniak P., Hertel A., Krupka G., Jagodziński P.P., Grygier M., Lesiak M. (2023). Circulating miRNA-451a and miRNA-328-3p as Potential Markers of Coronary Artery Aneurysmal Disease. Int. J. Mol. Sci..

[95] Li J.-Z., Li J., Liu B.-Z. (2019). MicroRNA-328-3p Inhibits Malignant Progression of Hepatocellular Carcinoma by Regulating MMP-9 Level. Eur. Rev. Med. Pharmacol. Sci..

[96] Ma W., Ma C., Zhou N., Li X., Zhang Y. (2016). Up- Regulation of miR-328-3p Sensitizes Non-Small Cell Lung Cancer to Radiotherapy. Sci. Rep..

[97] Srivastava A.K., Banerjee A., Cui T., Han C., Cai S., Liu L., Wu D., Cui R., Li Z., Zhang X. (2019). Inhibition of miR-328–3p Impairs Cancer Stem Cell Function and Prevents Metastasis in Ovarian Cancer. Cancer Res..

[98] Yan T., Ye X.-X. (2019). MicroRNA-328-3p Inhibits the Tumorigenesis of Bladder Cancer through Targeting ITGA5 and Inactivating PI3K/AKT Pathway. Eur. Rev. Med. Pharmacol. Sci..

[99] Baier S.R., Nguyen C., Xie F., Wood J.R., Zempleni J. (2014). MicroRNAs Are Absorbed in Biologically Meaningful Amounts from Nutritionally Relevant Doses of Cow Milk and Affect Gene Expression in Peripheral Blood Mononuclear Cells, HEK-293 Kidney Cell Cultures, and Mouse Livers123. J. Nutr..

[100] Zhang L., Hou D., Chen X., Li D., Zhu L., Zhang Y., Li J., Bian Z., Liang X., Cai X. (2012). Exogenous Plant MIR168a Specifically Targets Mammalian LDLRAP1: Evidence of Cross-Kingdom Regulation by microRNA. Cell Res..

[101] Van Herwijnen M.J.C., Driedonks T.A.P., Snoek B.L., Kroon A.M.T., Kleinjan M., Jorritsma R., Pieterse C.M.J., Hoen E.N.M.N.-‘t., Wauben M.H.M. (2018). Abundantly Present miRNAs in Milk-Derived Extracellular Vesicles Are Conserved Between Mammals. Front. Nutr..

[102] Izumi H., Kosaka N., Shimizu T., Sekine K., Ochiya T., Takase M. (2012). Bovine Milk Contains microRNA and Messenger RNA That Are Stable under Degradative Conditions. J. Dairy Sci..

[103] Pieters B.C.H., Arntz O.J., Bennink M.B., Broeren M.G.A., van Caam A.P.M., Koenders M.I., van Lent P.L.E.M., van den Berg W.B., de Vries M., van der Kraan P.M. (2015). Commercial Cow Milk Contains Physically Stable Extracellular Vesicles Expressing Immunoregulatory TGF-β. PLoS ONE.

[104] Abou el qassim L., Martínez B., Rodríguez A., Dávalos A., López de las Hazas M.-C., Menéndez Miranda M., Royo L.J. (2023). Effects of Cow’s Milk Processing on MicroRNA Levels. Foods.

[105] Li N., Miao Y., Shan Y., Liu B., Li Y., Zhao L., Jia L. (2017). MiR-106b and miR-93 Regulate Cell Progression by Suppression of PTEN via PI3K/Akt Pathway in Breast Cancer. Cell Death Dis..

[106] Khorraminezhad L., Rudkowska I. (2022). Dairy Product Intake Modifies MicroRNA Expression among Individuals with Hyperinsulinemia: A Post-Intervention Cross-Sectional Study. Lifestyle Genom..

[107] Hlavna M., Raudenska M., Hudcova K., Gumulec J., Sztalmachova M., Tanhäuserova V., Babula P., Adam V., Eckschlager T., Kizek R. (2012). MicroRNAs and Zinc Metabolism-Related Gene Expression in Prostate Cancer Cell Lines Treated with Zinc(II) Ions. Int. J. Oncol..

[108] Shkembi B., Huppertz T. (2021). Influence of Dairy Products on Bioavailability of Zinc from Other Food Products: A Review of Complementarity at a Meal Level. Nutrients.

[109] Iqbal M.A., Ali A., Hadlich F., Oster M., Reyer H., Trakooljul N., Sommerfeld V., Rodehutscord M., Wimmers K., Ponsuksili S. (2021). Dietary Phosphorus and Calcium in Feed Affects miRNA Profiles and Their mRNA Targets in Jejunum of Two Strains of Laying Hens. Sci. Rep..

[110] Su Q., Liu Y., Lv X.-W., Dai R.-X., Yang X.-H., Kong B.-H. (2020). LncRNA TUG1 Mediates Ischemic Myocardial Injury by Targeting miR-132-3p/HDAC3 Axis. Am. J. Physiol. Heart Circ. Physiol..

[111] Gong X., Huang M., Chen L. (2022). Mechanism of miR-132-3p Promoting Neuroinflammation and Dopaminergic Neurodegeneration in Parkinson’s Disease. eNeuro.

[112] DiMarco M., Zhao H., Boulicault M., Richardson S.S. (2022). Why “Sex as a Biological Variable” Conflicts with Precision Medicine Initiatives. Cell Rep. Med..

[113] Legato M.J., Johnson P.A., Manson J.E. (2016). Consideration of Sex Differences in Medicine to Improve Health Care and Patient Outcomes. JAMA.

[114] Miller V.M., Rocca W.A., Faubion S.S. (2015). Sex Differences Research, Precision Medicine, and the Future of Women’s Health. J. Women’s Health.

[115] Christou E.A.A., Banos A., Kosmara D., Bertsias G., Boumpas D. (2019). Sexual Dimorphism in SLE: Above and beyond Sex Hormones. Lupus.

[116] Deny M., Popotas A., Hanssens L., Lefèvre N., Arroba Nuñez L.A., Ouafo G.S., Corazza F., Casimir G., Chamekh M. (2023). Sex-Biased Expression of Selected Chromosome x-Linked microRNAs with Potent Regulatory Effect on the Inflammatory Response in Children with Cystic Fibrosis: A Preliminary Pilot Investigation. Front. Immunol..

[117] Sharma S., Eghbali M. (2014). Influence of Sex Differences on microRNA Gene Regulation in Disease. Biol. Sex Differ..

[118] Pinheiro I., Dejager L., Libert C. (2011). X-Chromosome-Located microRNAs in Immunity: Might They Explain Male/Female Differences? The X Chromosome-Genomic Context May Affect X-Located miRNAs and Downstream Signaling, Thereby Contributing to the Enhanced Immune Response of Females. BioEssays News Rev. Mol. Cell. Dev. Biol..

[119] Cui C., Yang W., Shi J., Zhou Y., Yang J., Cui Q., Zhou Y. (2018). Identification and Analysis of Human Sex-Biased MicroRNAs. Genom. Proteom. Bioinform..

[120] Liu Y., Shi X., Xu B., Wang Z., Chen Y., Deng M. (2023). Differential Expression of Plasma-derived Exosomal miRNAs in Polycystic Ovary Syndrome as a Circulating Biomarker. Biomed. Rep..

[121] Zhong S., Borlak J. (2024). Sex Differences in the Tumor Promoting Effects of Tobacco Smoke in a cRaf Transgenic Lung Cancer Disease Model. Arch. Toxicol..

[122] Malmhäll C., Weidner J., Rådinger M. (2020). MicroRNA-155 Expression Suggests a Sex Disparity in Innate Lymphoid Cells at the Single-Cell Level. Cell. Mol. Immunol..

